# Inflammation in Sleep Debt and Sleep Disorders

**DOI:** 10.1155/2015/343265

**Published:** 2015-03-25

**Authors:** Leila Kheirandish-Gozal, David Gozal, Jean-Louis Pépin

**Affiliations:** ^1^Section of Pediatric Sleep Medicine, Department of Pediatrics, Pritzker School of Medicine, Biological Sciences Division, The University of Chicago, Chicago, IL 60637, USA; ^2^INSERM U 1042, HP2 Laboratory, Faculté de Médecine, Université Grenoble Alpes, 38042 Grenoble, France; ^3^CHU and Hôpital A. Michallon, Pôle Thorax et Vaisseaux, 38043 Grenoble, France

Not only sleep is an essential physiological function, but also plays important roles in promoting growth, maturation, and overall health. There is increasing interest regarding the impact of sleep and its disorders on the regulation of inflammatory processes and end-organ morbidities, particularly in the context of neurocognitive, metabolic, and cardiovascular diseases and their complications [1–6]. Furthermore, jetlag and other perturbations of the circadian clock have also been linked to the regulation of fundamental regulatory properties underlying inflammatory processes and metabolic homeostasis [7, 8].

Sleep disorders such as obstructive sleep apnea syndrome (OSAS), a highly prevalent health problem across the age spectrum, are epidemiologically and mechanistically linked to metabolic deregulation. In the last decade, the emergence of increasing obesity rates has further led to remarkable increases in the prevalence of OSAS, along with more prominent neurocognitive, behavioral, cardiovascular, and metabolic morbidities [2, 9].

Although the underlying mechanisms leading to OSAS-induced morbidities are likely multifactorial and remain to be fully elucidated, activation of inflammatory pathways by OSAS has emerged as an important pathophysiological component of the end-organ injury associated with this disorder. To this effect, it would appear that OSAS could be viewed as a chronic, low-grade inflammatory disorder. Furthermore, the concurrent presence of obesity and OSAS poses a theoretically increased risk of OSAS-related complications.

In this special issue, studies covering aspects of inflammatory processes as they relate to sleep curtailment, sleep perturbation, or sleep disorders, such as OSAS, are presented and further reinforce the conceptual framework that sleep is a homeostatic regulator of inflammatory pathways and that perturbations in either sleep or inflammation will reciprocally affect each other.

M. S. Thimgan et al. report on the presence of elevated inflammatory gene transcripts such as prostaglandin-endoperoxide synthase 2 in the saliva of patients with OSAS and excessive daytime sleepiness (EDS), as well as in those with EDS, but in the absence of OSAS. M. L. Fung further elaborates on the potential contributions of the carotid body and other peripheral chemoreceptors to the recruitment of inflammatory pathways in the context of perturbed sleep and OSAS. The paper by L. Poulain et al. describes how an integral component of OSAS, namely, intermittent hypoxia, recruits TLR4 mechanisms that propagate inflammatory processes in both visceral adipose tissues and large blood vessels, ultimately promoting the emergence of insulin resistance. As a corollary of such processes, A. Gileles-Hillel et al. show that obese children with OSAS display evidence of enhanced levels of a selected array of inflammatory biomarkers in the circulation. Y. Nachalon et al. further demonstrate that treatment of OSAS by surgical removal of tonsils and adenoids leads to a reduction in the plasma levels of C-reactive protein (CRP), along with improved somatic growth. Furthermore, I. Bouloukaki et al. describe the association between the presence of erectile dysfunction in patients with severe OSAS and the concomitant elevation of inflammatory markers such as CRP, as well as tumor necrosis factor-*α*, interleukin-6, and interleukin-8. Finally, E. Paschetta et al. draw the attention to the potential inflammatory pathways that underlie the causal association between OSAS and liver injury, particularly nonalcoholic steatohepatitis.

We hope that the readers of this special issue will find the studies presented here not only interesting, but also further stimulating discussion and promoting the incorporation of the conceptual frameworks developed herein into the clinical, research, and educational realms.

*Leila Kheirandish-Gozal* *Leila Kheirandish-Gozal* *David Gozal* *David Gozal* *Jean-Louis Pépin* *Jean-Louis Pépin*
